# Editorial: Genomic Colocalization and Enrichment Analyses

**DOI:** 10.3389/fgene.2020.617876

**Published:** 2021-01-26

**Authors:** Chakravarthi Kanduri, Geir Kjetil Sandve, Eivind Hovig, Subhajyoti De, Ryan M. Layer

**Affiliations:** ^1^Department of Informatics, University of Oslo, Oslo, Norway; ^2^Department of Tumor Biology, Institute for Cancer Research, Radium Hospital, Oslo University Hospital, Oslo, Norway; ^3^Rutgers Cancer Institute of New Jersey, New Brunswick, GA, United States; ^4^Computer Science Department, University of Colorado, Boulder, CO, United States; ^5^BioFrontiers Institute, University of Colorado, Boulder, CO, United States

**Keywords:** colocalization analyses, bioinformatics, genomics, genome annotation, computational biology, enrichment analyses, co-occurrence analyses, genomic overlap

## Introduction

To decipher the molecular basis of health and disease, profiling multiple molecular modalities is a common practice [e.g., genetic variation, transcription, chromatin accessibility, epigenomic marks, binding sites, and three dimensional (3D) genome architecture]. Most of these molecular assays generate lists of genomic loci that are relevant to the trait/phenotype under investigation. Functional interpretation of these lists is often carried out through colocalization and enrichment analyses (Kanduri et al., 2018), which is akin to gene ontology/pathway analysis for lists of genes. A wide range of tools and methodologies have been developed over the past decade to perform colocalization and enrichment analyses of genomic regions. Given the availability and continuous generation of massive high resolution, cell-specific public datasets (e.g., ENCODE, RoadMap Epigenomics, GTEx, and BLUEPRINT), both existing and novel colocalization/enrichment analysis strategies will continue to generate new knowledge in our understanding of the molecular basis of health and disease. To highlight current research demonstrating the utility of colocalization/enrichment analysis, we invited contributions for a special Research Topic. The received contributions in this article collection include a comprehensive literature review, tools that extend the state-of-the-art methodology and enhance the user convenience in performing colocalization/enrichment analyses, and applied work that demonstrates the utility of colocalization/enrichment analyses.

## Literature Review Summarizing How Colocalization/Enrichment Analyses Have Aided the Functional Interpretation of GWAS Findings

Cano-Gamez and Trynka provide a detailed overview of how various strategies, especially enrichment and colocalization analysis, have aided in the interpretation of the findings of genome-wide association studies (GWAS). Specifically, the authors summarized single nucleotide polymorphism (SNP) enrichment analysis and statistical colocalization analysis. SNP enrichment analysis is one way to identify the tissue/cell types that are relevant for a disease by integrating either genome-wide-significant or a full set of assayed SNPs with molecular annotation tracks (e.g., either gene expression or chromatin accessibility). Once the relevant tissue/cell types are identified, further refined analysis using similar statistical analysis methods could disentangle the enrichments in highly similar cell types (e.g., to differentiate between cell states). Statistical colocalization analysis is one way to interpret novel GWAS findings by linking GWAS findings with likely target genes. This can be achieved by integrating GWAS signal with eQTL data to evaluate whether the same variant is causal in both GWAS and eQTL studies. In addition to summarizing the knowledge and strategies of the SNP enrichment and colocalization analysis, the authors have also provided perspectives on how the state-of-the-art technologies (e.g., single cell sequencing, genome editing) could be utilized in the future for the interpretation of GWAS findings.

## Tool that Extends the State-of-the-Art

One of the applications of colocalization analysis is for the interpretation of the functions of non-coding genomic regions. GREAT (McLean et al., 2010) and many other similar tools assign a regulatory domain for each gene that extends user-customizable distance both upstream and downstream to the transcription start site (TSS) of that gene. The regions of DNA binding events (both proximal and distal) are then assigned to genes, and subsequent statistical testing akin to traditional gene ontology analysis is performed to aid the functional interpretation. In this special collection, a novel method titled ProxReg (Lee et al.) complements the current state-of-the-art methods to aid the functional interpretation of non-coding regions by extending the methodology to not only test the proximity to TSS, but also to enhancers. The authors show that ProxReg provides additional insights into the regulatory mechanisms and binding tendencies of transcription factors (e.g., cell-specific regulatory mechanisms of the same TF by binding at promoters in one cell type and binding at enhancers in another cell type).

## Tools that Enhance the User Convenience in Performing Colocalization/Enrichment Analyses

### EpiColoc

One of the arduous tasks when using colocalization analysis tools to test/generate hypotheses is the need to carefully curate a collection of reference genomic tracks that are annotated thoroughly. Existing tools provide carefully curated collections of reference track collections (e.g., see Sheffield and Bock, 2016; Simovski et al., 2017; Layer et al., 2018); but epiColoc (Zhou et al.) published in this special issue takes a step further in this direction, and provides large collections of curated genomic tracks (44,385 bulk/single cell genomic tracks across 53 human cell/tissue types). The curated data span across transcriptional regulators, histone modifications, chromatin accessibility, transcriptional events, and chromatin segmentation data.

### LD-Annot

To perform any colocalization or enrichment analysis that involves SNPs, it is desirable to include statistically significant SNPs and all SNPs that are in tight linkage disequilibrium (LD) with them. Often, subsequent enrichment analyses are carried out on reference genome annotations that are overlapping the LD blocks. LD-annot provides a convenient wrapper around the popular PLINK tool (Chang et al., 2015) that computes LD between the genotypes of a given dataset and uses that information to intersect and extract the reference genome annotations overlapping the LD blocks.

## Applied Work

The study by Cresswell and Dozmorov, which includes a novel method titled TADcompare, demonstrates the utility of colocalization/enrichment analyses in aiding the functional interpretation of genomic regions with unknown biological significance. TADcompare is a method specifically developed to identify the changes in interacting domains (one of the features of three-dimensional genome architecture) and compare them across different conditions. One of the main challenges for TADcompare (as noted by the authors) was that no ground truth exists for boundaries of interacting domains, making it difficult to quantify the identified boundaries' biological relevance. To tackle this challenge, the authors of TADcompare used a range of colocalization analyses of epigenomic annotations and also a colocalization-based gene ontology enrichment analysis to determine whether the known genomic features that are characteristic of interacting domains and boundaries are enriched proximal to the identified boundaries and if that is different than background (non-boundaries).

The study by Ronzio et al. presents a new pipeline based on colocalization/enrichment analyses to identify regulatory modules of transcription factors (TFs) and TF recruitment rules. Instead of requiring overlap between a pair of ChIP-seq tracks, a proximity-based test statistic is suggested to quantify colocalization. The significance (*p*-value) is computed according to either hypergeometric or Poisson distribution. One possibility in the pipeline is to convert the *p*-values into scores and perform clustering analysis between the scores of multiple experiments to visualize potential regulatory modules. Further, motif enrichment analysis either relative to the whole accessible DNA or selected windows (e.g., upstream/downstream regions) could be performed for a pair of TFs. One could draw inferences on the recruitment patterns based on the observed pattern of motif enrichment (motifs for both TFs enriched or only one of them or none). Construction of the background sets in both colocalization analysis and motif enrichment analysis using alternative definitions (e.g., focusing only relative to enhancers/promoters) would allow one to identify specific regulatory modules.

The study by Tan et al. extends the traditional GSEA approach to establish associations between chemical-associated gene sets and gene expression in colorectal and rectal cancers. In the absence of a reliable tool to simulate CNVs from whole-exome sequencing data, Xing et al. developed the SECNVs tool which can generate CNVs with multiple customizable parameter options to mimic realistic CNVs from experimental data. The simulated CNV datasets could be utilized to explore the patterns of enrichment of CNVs in various contexts. For example, earlier others (Alexandrov et al., 2020; Singh et al., 2020) analyzed the patterns of enrichment of somatic mutations in tumor genomes and associated mutational signatures in their (epi)genomic contexts to infer their likely etiologies during tumorigenesis.

Overall, this Research Topic summarizes and showcases some of the existing and novel ways of utilizing genome colocalization/enrichment analyses to study a wide range of genetics and genomic research questions. The methods and tools published in this Research Topic extend the state of the art and enhance user convenience in performing genomic colocalization/enrichment analysis. With the continuous increase in the generation of genomic/epigenomic datasets, the interpretation of the resulting genomic regions becomes vital; we expect that the methodological principles of genomic colocalization/enrichment analysis will be utilized in many innovative ways in the future to further aid the functional interpretation of genomics datasets.

## Author Contributions

CK wrote the manuscript. GS, EH, SD, and RL edited the manuscript. All authors contributed to the article and approved the submitted version.

## Conflict of Interest

The authors declare that the research was conducted in the absence of any commercial or financial relationships that could be construed as a potential conflict of interest.
